# Attitudes, practices, and priority of HIV screening and testing among clinical providers in Transylvania and Moldavia, Romania

**DOI:** 10.1186/s12913-019-4823-5

**Published:** 2019-12-16

**Authors:** Cabiria M. Barbosu, Amanda Radulescu, Carmen Manciuc, Erin Muir, Brooke A. Levandowski, Timothy Dye

**Affiliations:** 1 University of Rochester School of Medicine and Dentistry, Rochester, New York 1464 USA; 2 “Iuliu Hatieganu” University of Medicine and Pharmacy, Cluj-Napoca, Romania; 3 “Gr.T.Popa” University of Medicine and Pharmacy Iași, Iasi, Romania

**Keywords:** Romania, HIV/AIDS, Screening, Testing, Transylvania, Moldavia

## Abstract

**Abstract:**

Screening and linkage to care are core, foundational strategies for HIV transmission prevention and for identifying People Living with HIV (PLHIV). In Romania – with an atypical experience in the HIV/AIDS epidemic – providing care for HIV+ patients identified early is a priority, though screening and testing can pose a challenge in some areas.

**Methods:**

A survey of 125 clinical providers to explore important dimensions of HIV/ AIDS clinical care was conducted in Transylvania and Moldavia, where clinicians identified poor/ latent screening as a major problem in providing timely care and in preventing the spread of disease. We analyzed determinants of offering HIV screening/testing to patients using Pearson Chi-square analysis and logistic regression. Logistic regression generated Odds Ratios (OR) to reflect the magnitude of association between the relevant variables, with 95% confidence interval (95% CI) indicating statistical range.

**Results:**

In total, 40.8% of providers did not provide HIV screening/testing to at least one segment of the population. Hospital-based providers were significantly more likely to offer HIV screening/testing to all segments than were non-hospital-based providers (58.1% v. 35.5%, respectively; *p* < .05). Providers located within institutions with screening/testing policies were more likely to offer such services to their patients (p < .05). Overall, 94.4% of providers indicated interest in more training around HIV screening/testing.

**Discussion:**

Reaching Romanian and global goals for reducing HIV require more timely screening and action based on positive status. Romanian clinicians are interested in expanding HIV screening/testing and are interested in participating in training that helps them feel more prepared to undertake this work.

## Background

The World Health Organization (WHO) estimates that 40% of people infected with HIV do not know their HIV status [1]. The Joint United Nations Program on HIV/AIDS (UNAIDS) 90–90-90 goal is to have 90% of people with HIV knowing their status, 90% of patients on antiretroviral therapy (ART) treatment, and 90% with viral suppression by 2020, with all three goals sequentially contingent [2]. The first 90% goal of having people know their status directly affects the latter two goals, the last of which means that 90% of People Living with HIV (PLHIV) are unlikely to transmit HIV. Ultimately, the goal of 90–90-90 is to end to the AIDS epidemic by 2020 with transmission rates rapidly dropping over the decade. The first step of this ambitious goal is to start with 90% of people knowing their status, emphasizing the importance of HIV screening and testing [3].

The WHO currently recommends that for people in areas with a generalized HIV epidemic, everyone in primary care settings (including children) be routinely offered testing, along with people in antenatal, tuberculosis (TB), sexually transmitted infection (STI), and harm reduction clinics. In regions with more concentrated epidemics, the WHO only recommends testing in high risk clinical settings [3]. Those considered high risk, such as partners of PLHIV, men who have sex with men (MSM), intravenous drug users (IDU), sex workers, and prisoners, should be tested at least once a year. The European Centers for Disease Control (ECDC) emphasizes the importance of testing these vulnerable groups at higher frequencies, and both the WHO and ECDC emphasize the importance of testing pregnant women in addition [3, 4].

If a person screens positive for HIV, the WHO recommends that s/he receive health education and counseling services and then be retested before starting ART [3]. Once on ART, chances of acquiring an HIV-related illness are greatly reduced, and quality of life improves [5]. HIV can still be transmitted, however many studies have shown that once a person knows his/her status, s/he reduces their risky behaviors, improving consistent condom usage, and not sharing needles [5]. Thus, screening and testing not only leads to increases longevity and quality of life for PLHIV, but it also decreases the odds of transmission through the reduction of risky behaviors and through the consistent use of ART.

Further, if someone screens HIV negative, there are still positive public health benefits since testing is an opportunity to provide educational materials about condoms, sharing needles, and pre-exposure prophylaxis (PrEP), and to take action to implement safe sexual behaviors [3]. Learning about HIV transmission and preventive strategies could enable high risk groups who seek testing out of concern they may have been exposed to HIV to implement preventive behaviors.

Unfortunately, the ECDC observes that there is, generally, complex perceptions of one’s risk, so people at high risk may not seek testing in the first place [4]. A further barrier to testing is also fear that a positive diagnosis will lead to stigma [4]. Providers can also form a barrier to offering HIV testing if they do not feel comfortable discussing the subject with their patients. All of these barriers are routed in a lack of awareness and understanding, which can be ameliorated with better education on how to take a sexual history and training on HIV management. However, many countries in Europe are burdened by a high proportion of late HIV diagnoses that result in increased mortality and morbidity, and more transmission within the population.

Romania has an atypical HIV/AIDS epidemic. Under the rule of a longstanding dictatorship in Romania (which ended in 1989), abortion and contraceptives were illegal unless a woman already had 5 children [6]. The goal of these inhibitory laws were to raise the birth rate in Romania, with the unintended consequence that infants were either abandoned voluntarily because they were unwanted, the family didn’t have resources to support them, or the mother died during childbirth [7]. These infants then entered institutionalized orphanages that were in poor condition, overcrowded, and understaffed [8]. These orphanages commonly injected infants with syringes of whole blood to boost immunity and to treat widespread malnutrition and anemia [9]. Yet, sterilization was challenged by faulty electricity and sterilizing syringes was not frequently implemented. With the fall of the Ceaușescu regime post-1989 reports of HIV/AIDS were made public and the extent of the epidemic was realized. CDC confirmed that 94% of positive HIV cases were found in children younger than 13 years old [10]. Only 4% of the 10,000 children diagnosed in the 1990s were estimated to have transmitted vertically, indicating that the contaminated syringes were largely responsible for the spread of HIV in Romania [11]. This epidemic created a cohort of HIV positive children, with 1/3rd now deceased, leaving the remaining two thirds to manage their HIV/AIDS for the rest of their lives [8]. Subsequent diagnosis and treatment for this large cohort of children (now in their mid-20s) has been inconsistent and incomplete, resulting in non-continuous treatment, poor access, and stigmatization [11, 12]. Thus, this cohort is at the center of Romania’s HIV/AIDS epidemic, characterized by uneven screening complicated by fear and stigma. Romania is one country that observes late diagnoses (defined as a CD4 count less than 350 cell/mm3); indeed, in 2017, 60% of people with new HIV diagnoses in Romania had less than 350 cell/mm3 [13]. This high percentage of late diagnoses contributes to the high number of deaths due to AIDS in Romania, since ART is not as effective for those diagnosed in a late stage [13].

In 2014 Romania approved a 2014–2020 National Public Health Strategy that included strategic objectives related to the AIDS response. However, the budget estimations are limited and HIV prevention are affected by the lack of funding [12]. HIV testing is performed in hospitals or private clinical specialized centers as a differential diagnosis, not as a routine testing, one of the reasons the HIV infection is diagnosed very late during the evolution [14, 15].

The purpose of this analysis is to examine HIV testing and screening practices of health care providers in two areas of Romania: Transylvania and Moldavia. Both are urban centers located at a distance from Bucharest, the capital city of Romania, and both areas are affected by the HIV/AIDS epidemic. Prior research on this group of health care providers found them receptive to using social media as a vehicle for clinical learning and support [16].

## Methods

We conducted an electronic survey of clinical providers (physicians, residents, nurses, others) in Transylvania and Moldavia, two regions of Romania. The instrument was an adaptation of the New York State Department of Health AIDS Institute’s Clinical Education Initiative’s Assessment, responsive to locally-stated priorities and streamlined to better understand clinical and training priorities in Romania [17]. Participants (physicians, nurses, dentists, psychologists, and social workers) were identified through a snowball/referral process originated in the HIV clinical hubs in Cluj-Napoca (Transylvania) and Iași (Moldavia).

A REDCap (Research Electronic Data Capture) [18] survey, using a cloud-based system, was deployed through sharing an invitation with a link to the survey to potential participants. The health care providers were asked if they routinely provide HIV screening and testing to their patients 13–18, 19–30, and over 30 years old. The instrument included a range of multiple answer questions about services they provide, their training interests and needs, their position, geographic location, and institutional type.

### Analysis

If respondents did not provide HIV screening and testing to any segment, they were classified as “Not Provide HIV Screening and Testing,” the primary endpoint for these analyses. Data were reduced to reflect “Physician” and “Non-physician” respondents, and “Hospital” and “Non-Hospital” respondents. Data were analyzed using Pearson Chi-square analysis and forward stepwise logistic regression [19]. Logistic regression generated Odds Ratios (OR) to reflect the magnitude of association between the relevant variables, with 95% confidence interval (95% CI) indicating statistical range. Data missing the primary endpoint (“Not Provide HIV Screening and Testing”) were excluded from analysis. SPSS 24.0 (IBM Corporation, 2016) was used for all quantitative analyses.

### Ethical review

The project was approved by both Iuliu Hatieganu University of Medicine and Pharmacy, Cluj- Napoca (UMF-Cluj) and Gr. T. Popa University of Medicine and Pharmacy, Iași (UMF- Iași) and by the University of Rochester’s Research Subjects Review Board. The project was performed in accordance with the ethical standards contained within the 1964 Declaration of Helsinki and its later amendments.

## Results

Overall, 125 participants responded to the assessment of which two-thirds (62.9%), lived in Transylvania, three-fourths (75%) work in hospitals and the vast majority (81.5%) were physicians (Table 1). Participants located in rural areas were less likely (though not significantly) to offer HIV screening and testing than were their urban counterparts. Further, no differences in HIV screening and testing were noted by whether or not the respondent was a physician, nor in which region (Transylvania or Moldavia) the respondent was located. Specialized organizations that provide HIV treatment were more likely to also offer screening and testing (*p* < .001), as were organizations that had written policy in place around HIV screening. Organizations with written policies on post-exposure prophylaxis (PEP) were also more likely to offer HIV screening. Indeed, the odds ratio remains statistically significant between having institutional policies on HIV screening and testing in place and offering screening and testing to all segments of the population, even after controlling for hospital setting (Adjusted OR: 11.1; 95% CI: 3.0, 41.8): the relationship between having institutional policies in place on HIV testing and screening was significantly associated with the likelihood that screening is offered to all population segments in both hospital (*p* < .05) and non-hospital (*p* < .001) settings (data not shown).

**Table 1:** HIV Screening and Testing by Selected Institutional and Respondent Characteristics, Transylvania and Moldavia Clinical Provider Assessment, 2017

	Total	No, Do Not Offer HIV Screening/ Testing	Yes, Offer HIV Screening/ Testing	Odds Ratio	
Characteristic	n (%)	n (%)	n (%)	OR (95% CI)	*p*-value
Hospital-based	93 (75.0)	33 (35.5)	60 (64.5)	Referent	0.027
Non-hospital based	31 (25.0)	18 (58.1)	13 (41.9)	2.5 (1.1, 5.8)	
Transylvania	78 (62.9)	18 (39.1)	28 (60.9)	Referent	
Moldavia	46 (37.1)	33 (42.3)	45 (57.7)	1.1 (0.5, 2.4)	0.728
Urban	117 (94.4)	46 (39.3)	71 (60.7)	Referent	Urban
Rural	7 (5.6)	5 (71.4)	2 (28.6)	3.9 (0.7, 20.7)	0.095
Physician Provider	101 (81.5)	41 (40.6)	60 (59.4)	Referent	
Non-physician Provider	23 (18.5)	10 (43.5)	13 (56.5)	1.1 (0.5, 2.1)	0.800
Organization provides treatment for HIV patients	60 (48.4)	16 (25.0)	48 (75.0)	Referent	
Organization does not provide treatment for HIV patients	64 (51.6)	35 (58.3)	25 (41.7)	4.2 (2.0, 9.0)	<.001
Organization has a written policy on testing for HIV	103 (83.1)	33 (32.0)	70 (68.0)	Referent	
Organization does not have a written policy on testing for HIV	21 (16.9)	18 (85.7)	3 (14.3)	12.7 (3.5, 46.3)	<.001
Organization has a written policy on Post-Exposure Prophylaxis (PEP)	72 (58.1)	20 (27.8)	52 (72.2)	Referent	
Organization does not have a written policy on Post-Exposure Prophylaxis (PEP)	52 (41.9)	31 (59.6)	21 (40.4)	3.8 (1.8, 8.2)	<.001

As shown in Table 2, 40.8% of the clinical providers did not provide HIV screening and testing to at least one segment of the population. Participants practicing in hospital settings were significantly more likely to offer HIV screening and testing to all segments than were participants from non-hospital settings (58.1% v. 35.5%, respectively; *p* < .05). Hospital-based participants were significantly more likely to offer HIV screening and testing to 13–18 year olds than their non-hospital-based counterparts (p < .05). Though hospital-based participants were also more likely to screen patients from other age groups than were non-hospital-based participants, these differences were not statistically significant.

**Table 2:** HIV Screening and Testing by Institutional Type, Transylvania and Moldavia Clinical Provider Assessment, 2017

	Total n (%)	Hospital n (%)	Non-hospital n (%)	Hospital OR (95% CI)	*p*-value
Provides HIV screening and testing to:					
13–18 year olds	77 (61.6)	63 (67.7)	14 (45.2)	2.6 (1.1, 5.9)	0.026
19–30 year olds	92 (73.6)	71 (76.3)	21 (65.6)	1.7 (0.7, 4.0)	0.244
Over 30 year olds	91 (72.8)	71 (76.3)	12 (62.5)	1.9 (0.8, 4.6)	0.137
Does not provide HIV testing to one or more groups above	51 (40.8)	33 (35.5)	18 (58.1)	2.5 (1.1, 5.8)	0.027

*OR* Odds ratio, *CI* Confidence interval

Shown in Table 3, the most common reasons indicated for not offering HIV screening and testing were that participants didn’t feel that screening was their responsibility (33.3%) and that the institutions where the participant worked didn’t require screening (33.3%). Some participants also indicated that they would prefer more training on HIV screening and testing (11.8%) and that there were other barriers in their organizations that prevented HIV screening and testing. Participants indicated that more training on HIV screening and testing (37.3%) and more training on how to talk with patients about HIV (31.4%) were priorities, as was providing more justification why testing is important (27.5%). When prompted around future training interests, 38.4% of providers were “somewhat interested” in more training around HIV screening and testing, and 56.0% of providers indicated they were “very interested” in such training (data not shown).

**Table 3:** Reasons for not offering HIV screening and testing and preferences for training and intervention, Transylvania and Moldavia Clinical Provider Assessment, 2017

Reason indicated for not screening and testing (among participants indicating they did not screen at least one group)	n (%) (*n* = 51)
It is not my responsibility	17 (33.3)
I don’t think all my patients are at risk	4 (7.8)
I feel uncomfortable asking some patients	3 (5.9)
I don’t think there is a good reason to screen	1 (2.0)
I need training in HIV testing	6 (11.8)
The organization I work for does not require that I do it	17 (33.3)
There are barriers in my organization that prevent me from doing so	6 (11.8)
What would help you recommend HIV testing to all your patients?	
More justification for why it is important	14 (27.5)
Training in HIV testing	19 (37.3)
Training in how to ask patients	16 (31.4)

## Discussion

Screening and testing for HIV is the cornerstone of ending the epidemic globally [3]. The WHO and UNAIDS promote universal access to knowledge of HIV status [20]. Early detection prolongs lives, prevents transmission, and reduces avoidable morbidity and mortality [21]. In the context of an unusual and atypical HIV/AIDS epidemic in Romania, a large portion of health care providers indicated that they do not routinely test and screen for HIV. Promoting greater access to knowledge of HIV status for Romanians will require deliberate policies and programs aimed at reducing the gaps that currently exist, gaps that miss identification of PLHIV and result in late presentation for care of people with advanced disease.

This study used snowball sampling to identify as many HIV providers as possible in two large Romanian cities. While this sampling strategy is limited to only obtaining the perspectives of those who were named, three authors are current or former HIV clinicians in Romania. Due to the small community of HIV clinicians and advance planning of obtaining clinician lists and email addresses, we feel this study was distributed to the majority of HIV clinicians in both cities. The results are subject to nonresponse bias.

This assessment indicates that screening within the hospital systems occur more frequently than in the non-hospital settings. Promoting HIV screening within the acute medical care environment of the hospital is an opportunity to identify PLHIV, perhaps secondary to other purposes for their hospital engagement. This hospital-level screening identifies PLHIV and links them with care, while also helping to prevent occupational exposure to HIV for health workers within that setting. A perhaps wider opportunity, however, exists with expanding HIV screening and testing efforts beyond the hospital, into primary care settings, and especially in rural towns and villages. HIV screening is not common in these areas, and given Romania’s unique experience with a cohort of HIV-exposed children now living as adults [22], screening efforts in rural areas could be an important element of prevention. Non-hospital sites of care seem particularly reluctant to screen teenagers; addressing the potential of this population to be exposed to HIV and remain undetected until late AIDS stage could form an important element of prevention for Romania.

Among the 23 European Union countries, Romania has more than 60% of late diagnosis (CD4 cell count < 350/mm3) suggesting that early infection is poorly diagnosed and evaluated [13] The new approaches of testing outside of conventional health facilities (hospitals) delivered within communities by trained medical staff and promoting indiviual testing as home sampling and self-testing should be considered [14].

The ECDC/WHO 2018 report shows that the new HIV diagnosis in Romania were heterosexual, MSM and IDU transmitted, and more than 60% were diagnosed late (CD4 cell count < 350/mm3) compared with the EU/EEA average of 49% late diagnosis [13]. In fact, a shocking 37% of IDUs are diagnosed with a CD4 cell count < 100/mm3. Thus, while Romania has achieved the first 90% goal, it is not surprising that it is falling short of 90% for meeting treatment needs, since those being diagnosed late cannot receive as effective treatment [23]. This suggests that achieving 90% of people knowing their status requires that people also need to know their status early, in order to progress towards the next 90% goal. Consequently, the new approaches of testing outside of hospitals and conventional health facilities delivered in rural areas and within communities by trained medical staff, and promoting indiviual self-testing as home sampling should be considered, as in Romania there are no policy or guidlines related to community based testing or self-testing [14, 24].

Further, specialized institutions that offer HIV treatment and have institutional policies on place on HIV screening and testing and also on PEP, are more likely to offer screening and testing to general population. Having institutional policies in place has been demonstrated elsewhere as an effective strategy for promoting HIV testing and screening [25] being considered best practice [21]. A substantial portion of the survey’s respondents who do not currently offer HIV screening and testing, indicated that their institutions do not require the implementation of such procedures, indicated that they do not feel it was their responsibility, or believed there were other institutional challenges that limited their ability to screen and test for HIV. A substantial prevention obstacle and also a profound public health problem represent the fact that in Romania the HIV testing is performed only in the hospital setting, as a differential diagnosis, and not performed as a routine test at the primary care/ family doctor office [14]. Addressing the institutional environment could help promote HIV testing and screening significantly [26].

Almost all providers indicated they were at least somewhat interested in more training around HIV screening and testing, and a substantial portion of providers who did not currently offer routine HIV screening and testing to at least one of the segmented populations indicated that more training on HIV screening and testing, more rationale for offering screening and testing, and training on how to talk with patients about HIV would help them recommend HIV screening to all their patients. These providers are also currently using social media platforms, and were interested in using social media technology for clinical learning and support [16]. Such training has been demonstrated elsewhere as an effective component of enhancing HIV screening, and could form a component of local and regional targeted continuing medical education, integration with medical training, and also in training of ancillary personnel [27].

Despite much progress, rates of HIV testing in Europe remains low. For example in Romania, HIV testing performed to TB patients is only 3.4%, testing in pregnant women is 39.2% and testing by request is 52%. Also criminalization remains a barrier to providing testing in some countries, including Romania [24].

## Conclusion

Providing targeted training to clinicians around HIV screening and testing – along with assistance to institutions to develop institutional policies around HIV-related screening, testing and care – could help improve identification of PLHIV before their disease progresses to an advanced stage. In Romania’s unique experience with an atypical HIV epidemiology and progression, such medical trainings could form an important part of the plan to end the epidemic by 2020.

## Data Availability

Interested parties may request de-identified copies of the data used in this analysis. Contact the primary author Monica_Barbosu@URMC.Rochester.edu for more information.

## Abbreviations

ECDC: The European Centers for Disease Control
IDU: intravenous drug users
MSM: Men who have Sex with Men
PEP: Post exposure prophylaxis
PLHIV: People Living with HIV/AIDS
PrEP: Pre exposure prophylaxis
STI: Sexually Transmitted Infection
TB: Tuberculosis
WH: World Health Organization

## References

[1] Joint United Nations Programme on HIV/AIDS. Prevention Gap Report. In: Vol 3. Geneva, Switzerland.2016: http://www.unaids.org/en/resources/documents/2016/prevention-gap..

[2] HIV/AIDS JUNPo. 90–90-90: An ambitious treatment target to help end the AIDS epidemic. *Geneva: UNAIDS.* 2014.

[3] World Health Organization. Consolidated guidelines on HIV testing services: 5Cs: consent, confidentiality, counselling, correct results and connection 2015. World Health Organization; 2015. https://apps.who.int/iris/handle/10665/179870. 26378328

[4] European Centres for Disease Control (2010). HIV testing: increasing uptake and effectiveness in the European Union.

[5] Branson BM, Handsfield HH, Lampe MA, et al. Revised recommendations for HIV testing of adults, adolescents, and pregnant women in health-care settings. MMWR Recomm Rep*.* 2006;55(14):1-CE-4. 16988643

[6] Dente K, Hess J (2006). Pediatric AIDS in Romania–a country faces its epidemic and serves as a model of success. Medscape Gen Med.

[7] Morrison L (2004). Ceausescu’s legacy: family struggles and institutionalization of children in Romania. J Fam Hist.

[8] Botescu A, Abagiu A, Mardarescu M, Ursan M. HIV/AIDS among injecting drug users in Romania. Report of a recent outbreak and initial response policies. Lisbon: European Monitoring Centre for Drugs and Drug Addiction; 2012. http://www.emcdda.europa.eu/publications/ad-hoc/2012/romania-hiv-update_en.

[9] Ruland CD, Finger W, Williamson N, et al. Adolescents: Orphaned and Vulnerable in the Time of HIV/AIDS. Family Health International, YouthNet Program Washington, DC; 2005.

[10] Kozinetz CA, Matusa R, Hacker CS (2006). Biologic and social determinants of sequelae and long-term survival of pediatric HIV in Romania. Ann Epidemiol.

[11] Kozinetz CA, Matusa R, Cazacu A (2001). The burden of pediatric HIV/AIDS in Constanta, Romania: a cross-sectional study. BMC Infect Dis.

[12] UNAIDS. Country Progress Report, 2015, Romania. 2016; http://www.unaids.org/sites/default/files/country/documents//file,94721,ru..pdf..

[13] European Centre for Disease Prevention and Control (ECDC). HIV/ AIDS Surveillance in Europe 2018. 2017 data; https://www.ecdc.europa.eu/en/publications-data/presentation-hivaids-surveillance-europe-2018-2017-data

[14] Rutã S, Cernescu C (2008). Influence of social changes on the evolution of HIV infection in Romania. Int J Environ Stud.

[15] Baylor International Pediatric AIDS Initiative. Romania. *Romania* 2019; https://bipai.org/romania..

[16] Manciuc C, Levandowski BA, Muir E, Radulescu A, Barbosu M, Dye TD (2018). Access to digital and social media among Romanian HIV/AIDS clinical providers. Glob Health Action.

[17] Barbosu CM, Perez-Ramos JG, Demment M (2017). 2511: use of an online provider learning community to assess clinical HIV/HCV/STDs-related training needs. J Clin Transl Sci.

[18] Harris PA, Taylor R, Thielke R, Payne J, Gonzalez N, Conde JG (2009). Research electronic data capture (REDCap)—a metadata-driven methodology and workflow process for providing translational research informatics support. J Biomed Inform.

[19] Hosmer Jr DW, Lemeshow S. Applied logistic regression. John Wiley & Sons; 2004.

[20] Organization WH. Statement on HIV testing and Counselling: WHO, UNAIDS re-affirm opposition to mandatory HIV testing. WHO, Geneva 2012;28.

[21] Centers for Disease Control and Prevention. Revised guidelines for HIV counseling, testing, and referral. *MMWR Recommendations and reports: Morbidity and mortality weekly report Recommendations and reports/Centers for Disease Control.* 2001;50(RR-19):1. 11718472

[22] Hersh BS, Popovici F, Jezek Z (1993). Risk factors for HIV infection among abandoned Romanian children. Aids.

[23] Granich R, Williams B, Montaner J, Zuniga JM (2017). 90-90-90 and ending AIDS: necessary and feasible. Lancet.

[24] HIV Testiong in Europe - Dublin Declaration Report 2017 https://www.ecdc.europa.eu/sites/default/files/documents/HIV%20testing.pdf

[25] Deblonde J, De Koker P, Hamers FF, Fontaine J, Luchters S, Temmerman M (2010). Barriers to HIV testing in Europe: a systematic review. Eur J Pub Health.

[26] Johns DM, Bayer R, Fairchild AL (2016). Evidence and the politics of deimplementation: the rise and decline of the “counseling and testing” paradigm for HIV prevention at the US Centers for Disease Control and Prevention. Milbank Q.

[27] Centers for Disease Control and Prevention. High-impact HIV prevention: CDC's approach to reducing HIV infections in the United States. 2011; https://www.cdc.gov/hiv/policies/hip/hip.html.

